# Immune-related diagnostic indicators and targeted therapies for COPD combined with NASH were identified and verified via WGCNA and LASSO

**DOI:** 10.3389/fimmu.2025.1514422

**Published:** 2025-02-28

**Authors:** Jianwei Hong, Zikai Xu, Fangrui Xu, Haifeng Wu, Jinxia Liu, Lishuai Qu

**Affiliations:** ^1^ Department of Gastroenterology, Affiliated Hospital of Nantong University, Medical School of Nantong University, Nantong, China; ^2^ Department of Respiratory and Critical Care Medicine, Affiliated Hospital of Nantong University, Medical School of Nantong University, Nantong, China; ^3^ Department of Medical Imaging, Affiliated Hospital of Nantong University, Medical School of Nantong University, Nantong, China; ^4^ Department of Emergency Medicine, Affiliated Nantong Hospital of Shanghai University (The Sixth People’s Hospital of Nantong), Nantong, Jiangsu, China

**Keywords:** NAFLD, COPD, inflammation, immunity, metabolism, diagnostic markers, WGCNA, machine learning

## Abstract

**Introduction:**

The incidence of chronic obstructive pulmonary disease (COPD) and non-alcoholic fatty liver disease (NAFLD) has increased significantly in past decades, posing a significant public health burden. An increasing amount of research points to a connection between COPD and NAFLD. This study aimed to identify the key genes of these two diseases, construct a diagnostic model, and predict potential therapeutic agents based on critical genes.

**Methods:**

NAFLD and COPD datasets were obtained from the GEO database, differential genes were identified by differential analysis and WGCNA, PPI networks were constructed and enriched for differential genes and COPD-associated secreted proteins, small molecule compounds were screened, and immune cell infiltration was assessed. Meanwhile, LASSO and RF further screened the essential genes, and finally, two key genes were obtained. Subsequently, the nomogram diagnostic model and lncRNA-miRNA-mRNA network were constructed based on these two core genes, subjected to drug prediction and GSEA enrichment analysis, and validated in an external cohort using qRT-PCR.

**Results:**

KEGG enrichment analysis indicated that the NF-kappa B and TNF signaling pathways may be associated with COPD and NASH co-morbidities. Ten small-molecule drugs associated with COPD and NASH were identified through cMAP analysis, including ansoprazole and atovaquone. In addition, we further identified the hub genes S100A9 and MYH2 for NAFLD and COPD by machine learning methods. The immune infiltration indicated that these two core genes might be involved in the immunomodulatory process of NASH by regulating the function or recruitment of specific immune cell types. A nomogram diagnostic model was constructed based on these two core genes. The AUC value for S100A9 was 0.887, for MYH2 was 0.877, and for the nomogram was 0.889, demonstrating excellent diagnostic efficacy. Two hundred fifty-four potential drugs targeting S100A9 and 67 MYH2 were searched in the DGIdb database. Meanwhile, the lncRNA-miRNA-mRNA network was constructed by predicting target miRNAs of biomarkers and further predicting lncRNAs targeting miRNAs. qRT-PCR analysis revealed that S100A9 was upregulated in both COPD and NAFLD, consistent with bioinformatic predictions, while MYH2 showed increased expression in COPD but decreased expression in NAFLD, diverging from the predicted downregulation in both diseases. These findings suggest that S100A9 serves as a common inflammatory marker for both diseases, whereas MYH2 may be regulated by disease-specific mechanisms, highlighting its potential for distinguishing COPD from NAFLD.

**Conclusion:**

The hub genes S100A9 and MYH2 in COPD and NASH were identified by various bioinformatics methods and a diagnostic model was constructed to improve the diagnostic efficiency. We also revealed some potential biological mechanisms of COPD and NASH and potential drugs for COPD-related NASH. Our findings provide potential new diagnostic and therapeutic options for COPD-associated NASH and may help reduce its prevalence.

## 1 Introduction

Prolonged airflow restriction with steady progression is a hallmark of chronic obstructive pulmonary disease (COPD), a prevalent and severe respiratory disease. The global incidence of COPD has risen dramatically, driven by population aging and the persistent increase in smoking. This condition remains a significant challenge for clinicians in the 21st century, imposing a substantial socio-economic and public health burden due to its high morbidity and mortality rates (1, 2). By 2030, COPD is predicted by the World Health Organization to rank third in terms of causes of mortality. Smoking and inhaling harmful particulate matter are known risk factors (3). In addition, the potential mechanisms by which inflammation, oxidative stress, and metabolic disorders contribute to the development of COPD are increasingly being studied (4).

Excessive intracellular fat buildup in hepatocytes as a result of the exclusion of alcohol and other known liver-damaging agents characterizes NAFLD, an acquired metabolic stress liver damage. This disease ranges from mild fatty liver to non-alcoholic steatohepatitis (NASH) and even to cirrhosis (5, 6). In the past decades, NAFLD has even emerged as the most prevalent chronic liver disease worldwide, affecting over 25% of adult subjects (7). Population predisposition to NAFLD exists, and it is typically linked to metabolic syndrome components, including hypertension, type 2 diabetes, and obesity (8).

Multiple studies have shown that NAFLD is associated with multi-system manifestations such as cardiovascular, renal, and endocrine (9, 10). Crosstalk between different organs leads to extrahepatic complications of NAFLD. Several investigations have shown that NAFLD is associated with several respiratory diseases, such as COPD (11–14). Viglino et al. screened 111 COPD patients with serum samples with various conditions. Hepatic steatosis, NASH, and hepatic fibrosis were noninvasively assessed in these patients using the FibroMax method, a diagnostic blood test that combines multiple biomarkers to estimate the degree of liver damage and fibrosis. The FibroMax method provides a non-invasive alternative to liver biopsy, offering a quantitative assessment of liver function and fibrosis. Eventually, they concluded that 41.4% of these 111 patients with COPD had moderate to severe steatosis, 36.9% had junctional NASH and another 61.3% had hepatic fibrosis (15). A study published by Lowie E.G.W. in the European Respiratory Journal found that hyperglycemia, dyslipidemia, and atherosclerosis were prevalent in patients with COPD, deepening our understanding of systemic comorbidities in patients with COPD (16). NAFLD is considered as one of the systemic comorbidities of COPD. We know that multiple factors involved in the progression of NAFLD, including oxidative stress, low-grade inflammation, low physical activity, insulin resistance, metabolic disorders, and lipid accumulation (17, 18). Lipid metabolism and inflammation and show a close association in the co-morbidity of COPD and NAFLD (18).

In recent years, bioinformatics and microarray technology have been rapidly developed, and a large amount of gene expression data has been made public, providing more help for researchers to study the diseases (19, 20). COPD and NAFLD seem to be two relatively independent pathophysiological processes, but more and more studies have shown a non-accidental link between these two diseases. Authoritative epidemiological and clinical evidence suggests that the COPD incidence in NASH is significantly higher than in the general population, with an increased risk of death (21). Therefore, screening for NASH in patients with COPD is necessary. Given that the gold standard for diagnosing NASH is the histological assessment of liver biopsies (22), an invasive procedure, there is an urgent need to find an efficient, minimally invasive, or non-invasive method for diagnosing NASH (23). Through the analysis, S100A9 and MYH2 were identified as two key biomarkers in this study. S100A9, a calcium-binding protein, is highly expressed in immune cells, particularly neutrophils, and typically forms a heterodimer with S100A8 (S100A8/S100A9), which plays a crucial role in regulating inflammatory processes. Members of the S100 protein family, including S100A8/S100A9, are also pivotal in the pathogenesis of NASH, especially in hepatic inflammatory cells (24, 25). Meanwhile, MYH2, which encodes the myosin heavy chain of type 2A fast-twitch muscle fibers, has been implicated in various hereditary myopathies. These conditions, which may follow autosomal dominant or recessive inheritance patterns, are characterized by muscle fiber atrophy (particularly type 2A fibers), impaired muscle function, and pathological features such as rimmed vacuoles, fiber-type variability, and fatty infiltration (26, 27). The combined role of S100A9 and MYH2 may offer novel insights into the diagnosis and treatment of COPD-associated NASH.

This study thus focuses on S100A9 and MYH2 to establish a blood-based, non-invasive diagnostic approach and to explore relevant therapeutic strategies, contributing to improved clinical management of COPD-associated NASH.

## 2 Materials and methods

### 2.1 Data collection and processing

**Table 1** provides comprehensive details about the datasets. Four NASH datasets, GSE24807, GSE48452, GSE66676, and GSE63067 were retrieved from the GEO database, comprising a total of 119 human liver tissue samples, including 58 normal liver tissues and 61 NASH tissues. These datasets were standardized by replacing negative values with zero and removing missing data. To address technical variability between datasets, batch effects were corrected using the ComBat function from the “sva” package. PCA was subsequently performed to evaluate sample distribution and confirm dataset harmonization for downstream analyses. Similarly, two COPD datasets, GSE38974 and GSE106986 were obtained from the GEO database, including 14 normal lung tissue samples and 37 COPD tissue samples. After aligning shared genes across datasets and replacing negative values with zero, batch effects were corrected using the same ComBat approach. Boxplots were generated before and after correction to assess the consistency of sample distributions and ensure proper data integration.

**Table 1:** Descriptive statistics of the GEO datasets.

GEO accession	Platform	Origin	Sample		Species
			Control	NAFLD	
GSE24807	GPL2895	liver	5	12	Homo sapiens
GSE48452	GPL11532	liver	12	14	Homo sapiens
GSE66676	GPL6244	liver	34	26	Homo sapiens
GSE63067	GPL570	liver	7	9	Homo sapiens

GEO accession	Platform	Origin	Sample		Species
			Control	COPD	
GSE38974	GPL4133	lung	9	23	Homo sapiens
GSE106986	GPL13497	lung	5	14	Homo sapiens

### 2.2 Differential gene analysis

We utilized the “Limma” package, a powerful R tool for differential analysis, to process the corrected NASH and COPD datasets and screen for differentially expressed genes (DEGs). DEGs, defined as genes exhibiting significant differences in expression levels under varying conditions, were filtered using thresholds of p ≤ 0.05 and |log2(fold change) | ≥ 0.585. The filtered DEGs were subsequently visualized using heat maps and volcano plots, generated with the “heatmap” and “ggplot2” packages in R software.

### 2.3 WGCNA

In the present analysis, we utilized the Weighted Gene Co-expression Network Analysis (WGCNA), a systems biology approach for studying gene expression data. We aimed to identify gene modules by building gene co-expression networks and investigating their relationship with various phenotypes or biological features. We selected a soft threshold power (β=3) as the weight to construct the gene co-expression networks. We then computed the weighted expression correlations to generate the topological overlap matrix (TOM), conducted hierarchical clustering analysis on the TOM, identified distinct gene modules, calculated the module eigenitem volume (ME) for each module, merged similar modules, and visualized the results using a heat map. Subsequently, we assessed the correlation of the module eigenvectors with the given traits and selected the module with the strongest correlation. Finally, we evaluated the significance of this module by determining the gene-module correlation (Module Membership, MM) and the gene-trait correlation (Gene Significance, GS).

### 2.4 COPD-secreted proteins

We downloaded 3946 genes encoding secreted proteins using The Human Protein Atlas database (https://www.proteinatlas.org/).

### 2.5 Construction of protein-protein interaction networks

To explore the connection between COPD-related secreted proteins and essential NASH genes, we created a protein-protein interaction (PPI) network using the “SRING” database (https://cn.string-db.org/), with a minimum interaction score of 0.4. We employed MCODE to identify the essential modules and chose those with the highest scores for further analysis.

### 2.6 Cellular function enrichment analysis

After identifying the differential genes and key modules, we delved into their biological functions and disease-causing mechanisms. We then conducted GO and KEGG enrichment analyses using the clusterProfiler package, encompassing BP, CC, and MF. A significance level of P<0.05 was applied to the enrichment analysis. Subsequently, we pinpointed significantly enriched pathways and functions and visualized them using the GOChord function. Moreover, we employed CMAP analysis to investigate the impact of small molecule compounds on gene expression and to uncover novel small molecule compounds associated with the target disease. These compounds could potentially serve as drug candidates for further research and development. In our study, we inputted the highest-scoring upregulated genes in the PPI network into the map database and identified ten small molecule compounds with top scores as potential drugs for treating NASH.

### 2.7 Machine learning

LASSO is a widely used statistical method for feature selection and regression analysis. Random forest is a machine learning algorithm based on integrated learning, which improves prediction accuracy and stability by integrating multiple decision trees. In our study, we initially utilized the LASSO algorithm to identify potential diagnostic genes from the shared genes of WGCNAs and DEGs. Subsequently, we employed the randomForest method to select disease signature genes, ranking them based on gene importance and using LASSO regression for feature selection. We then conducted cross-validation to screen potential diagnostic genes for intersection analysis, aiming to obtain the most effective model for identifying and improving the accuracy of diagnosis and treatment.

To create visual representations of predictive models’ results and probabilities, we used logistic regression and the lm function. We then constructed Nomogram plots using the nomogram function. The pROC package helped us calculate and plot the ROC curve, which we used to assess the model’s performance by calculating the area under the ROC curve (AUC) and 95% confidence interval (CI) values. Higher AUC values indicate better predictive ability. We also plotted calibration curves to compare predicted probabilities with actual incidences. Additionally, we performed DCA analysis to evaluate the effectiveness of the Nomogram model across different probability thresholds.

### 2.8 lncRNA-miRNA-mRNA network construction

The miRWalk database was used to predict biomarker target miRNAs. The ENCORI database was then used to indicate the lncRNAs targeting miRNAs. Cytoscape (version 3.8.2) was used to build lncRNA-miRNA-mRNA networks.

### 2.9 Single gene enrichment analysis GSEA

GSEA was conducted separately for the essential genes MYH2 and S100A9. To thoroughly examine the main pathways linked to the development of COPD and NASH. The dataset used as a reference was “h.all.v2023.2.Hs.symbols.gmt” and was acquired from MSigDB (28). The screening parameters of P< 0.05 and FDR< 0.25 were used to identify routes that showed significant enrichment. These pathways were then displayed using the “Enrichment Map” software package.

### 2.10 qRT-PCR

At the hospital, 75 blood samples were obtained from patients: 25 COPD patients, 25 NAFLD patients, and 25 healthy individuals. **Table 2** summarizes the clinical data collected from the patients. Each volunteer gave informed consent to use their serum in our study. We extracted total RNA using the Trizol method and performed concentration measurements, followed by reverse transcription. Next, qRT-PCR was performed using the cDNA as a template. Ultimately, the target gene’s expression data was normalized using GAPDH as the internal reference gene, and 2 - ΔΔCt was used to determine the target gene’s relative expression. The following primer sequences are used in this experiment:

**Table 2:** Patient baseline demographic and clinical characteristics.

Variables	Overall	COPD	NAFLD	Normal	p-value
	N = 75^1^	N = 25^1^	N = 25^1^	N = 25^1^	
**Age (years), Median (Q1, Q3)**	65.00 (61.00, 70.00)	65.00 (61.00, 68.00)	64.00 (61.00, 67.00)	65.00 (60.00, 73.00)	0.4822
**Gender, n (%)**					0.6873
Male	40.00 (53.33%)	15.00 (60.00%)	12.00 (48.00%)	13.00 (52.00%)	
Female	35.00 (46.67%)	10.00 (40.00%)	13.00 (52.00%)	12.00 (48.00%)	
**Height (cm), Mean ± SD**	165.33 ± 7.06	166.20 ± 7.65	165.56 ± 6.18	164.24 ± 7.40	0.2432
**Weight (kg), Mean ± SD**	66.23 ± 11.15	61.62 ± 11.87	71.20 ± 10.48	65.88 ± 9.21	0.0272
**BMI, Mean ± SD**	24.23 ± 3.81	22.29 ± 3.99	25.95 ± 3.28	24.45 ± 3.32	0.0022
**Hypertension, n (%)**					0.0303
No	45.00 (60.00%)	19.00 (76.00%)	10.00 (40.00%)	16.00 (64.00%)	
Yes	30.00 (40.00%)	6.00 (24.00%)	15.00 (60.00%)	9.00 (36.00%)	
**Diabetes, n (%)**					<0.001^3^
No	49.00 (65.33%)	18.00 (72.00%)	6.00 (24.00%)	25.00 (100.00%)	
Yes	26.00 (34.67%)	7.00 (28.00%)	19.00 (76.00%)	0.00 (0.00%)	
**Current Smoking Status, n (%)**					0.0053
No	55.00 (73.33%)	24.00 (96.00%)	17.00 (68.00%)	14.00 (56.00%)	
Yes	20.00 (26.67%)	1.00 (4.00%)	8.00 (32.00%)	11.00 (44.00%)	
**Ex-smoker Status, n (%)**					>0.999^3^
No	42.00 (56.00%)	14.00 (56.00%)	14.00 (56.00%)	14.00 (56.00%)	
Yes	33.00 (44.00%)	11.00 (44.00%)	11.00 (44.00%)	11.00 (44.00%)	
**Alcohol Consumption, n (%)**					0.5183
No	57.00 (76.00%)	17.00 (68.00%)	`	20.00 (80.00%)	
Yes	18.00 (24.00%)	8.00 (32.00%)	5.00 (20.00%)	5.00 (20.00%)	
**FEV1 predicted, Median (Q1, Q3)**	47.90 (36.80, 58.20)	47.90 (36.80, 58.20)	NA (NA, NA)	NA (NA, NA)	
**FEV1/FVC, Median (Q1, Q3)**	50.63 (37.63, 57.02)	50.63 (37.63, 57.02)	NA (NA, NA)	NA (NA, NA)	
LAMA Usage, n (%)					
Not using	25.00 (100.00%)	25.00 (100.00%)	0.00 (NA%)	0.00 (NA%)	
**iCS.LABA Usage , n (%)**					>0.999^4^
Not using	24.00 (96.00%)	24.00 (96.00%)	0.00 (NA%)	0.00 (NA%)	
Using	1.00 (4.00%)	1.00 (4.00%)	0.00 (NA%)	0.00 (NA%)	
**iCS.LABA.LAMA Usage, n (%)**					>0.999^4^
Not using	1.00 (4.00%)	1.00 (4.00%)	0.00 (NA%)	0.00 (NA%)	
Using	24.00 (96.00%)	24.00 (96.00%)	0.00 (NA%)	0.00 (NA%)	
**Blood glucose (mmol/L), Median (Q1, Q3)**	5.50 (4.90, 7.20)	5.30 (4.60, 5.50)	7.30 (5.90, 8.70)	5.20 (4.70, 6.40)	<0.001^2^

^1^Median (IQR) or Mean ± SD or Frequency (%).

^2^Kruskal-Wallis rank sum test.

^3^Pearson's Chi-squared test.

^4^Fisher's exact test.

S100A9: Forward: 5′-CTGTGTGGCTCCTCGGCTTTG-3′; Reverse: 5′-TGGTGGAAGGTGTTGATGATGGTC-3′.

MYH2: Forward: 5′-GCCAACTTCCAGAAGCCCAAGG-3′; Reverse: 5′-CAGTCCAACCACGGTCTCATTCAG-3′.

GAPDH: Forward: 5′-CAGGAGGCATTGCTGATGAT -3′; Reverse: 5′- GAAGGCTGGGGCTCATTT-3′.

## 3 Results

### 3.1 Data acquisition

**Figure 1:**
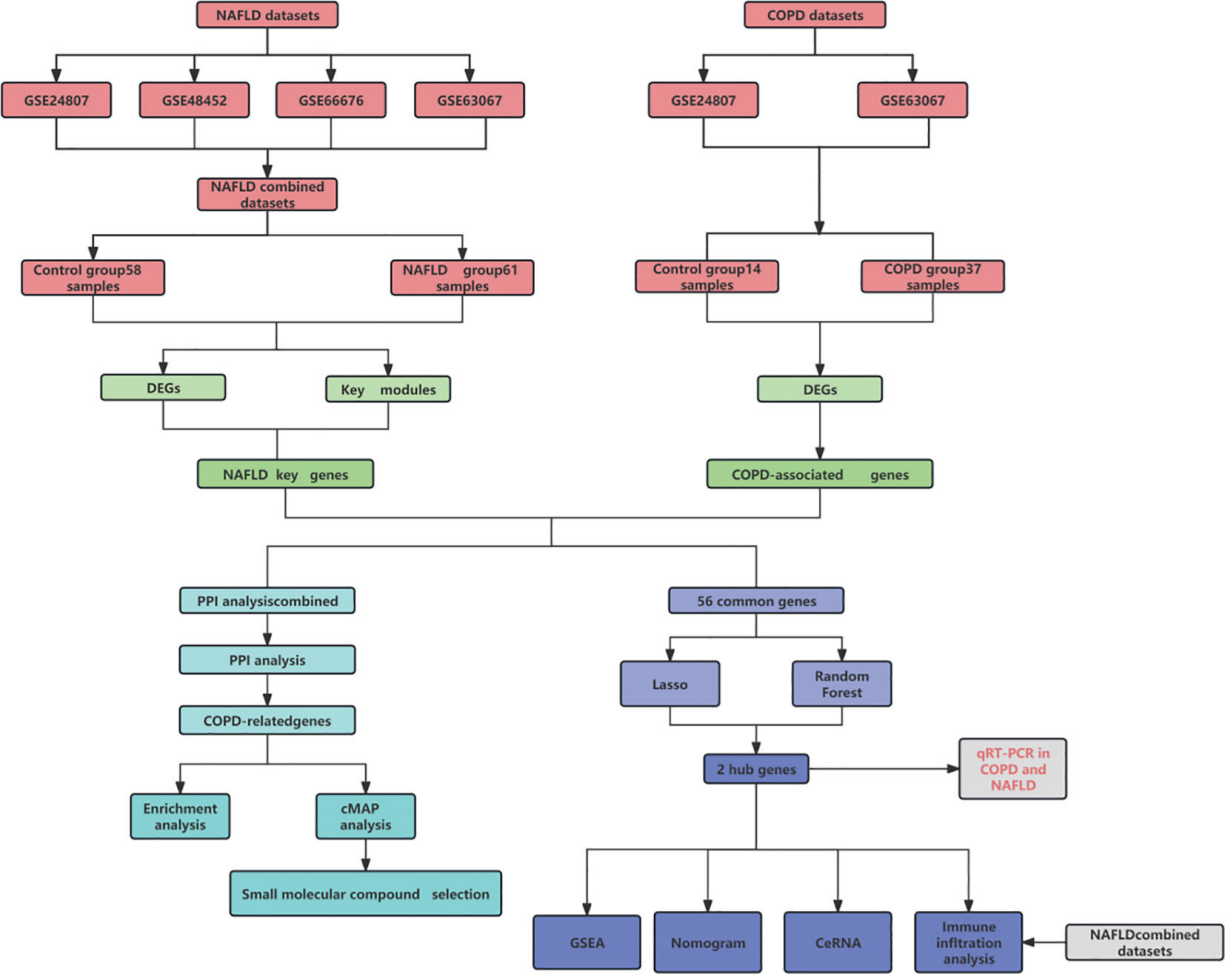
Pattern diagram of this study.

**Figure 2:**
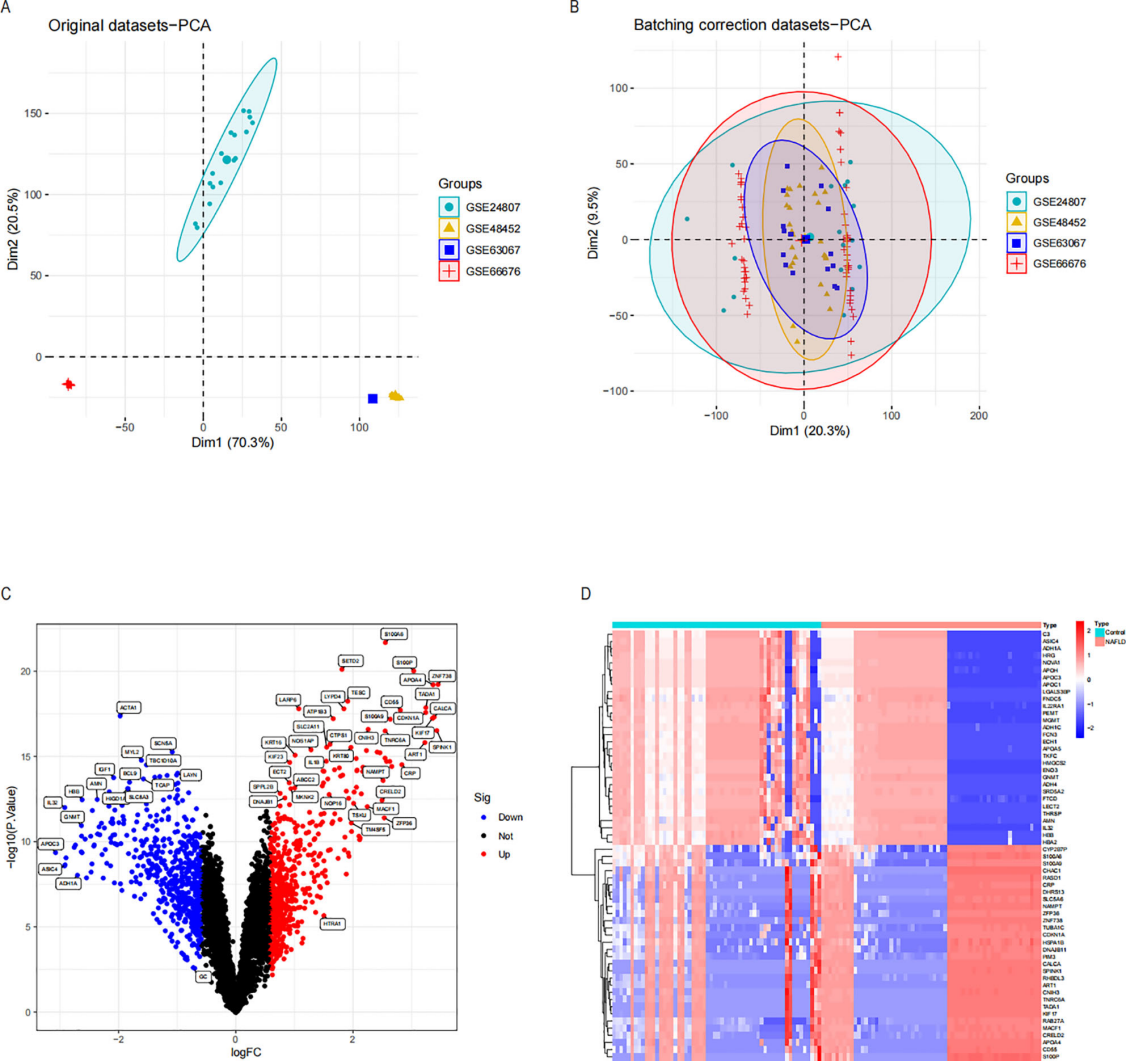
Differential expression analysis and principal component analysis of the NASH dataset were conducted before and after the de-batch effect. **(A)** PCA on four sets of raw NASH data before batch effect correction. **(B)** PCA following batch effect correction on the combined NASH dataset. **(C)** Volcano plots of DEGs in the integrated NASH dataset, which represent NASH. Genes that are up-regulated are shown by red dots and those that are down-regulated by blue dots. **(D)** A heatmap displaying the DEGs in the NASH dataset that are up-and down-regulated. NASH, Non-alcoholic steatohepatitis; PCA, Principal Component Analysis; DEGs, Differentially Expressed Genes.

**Figure 1** displays the schematic diagram. We have downloaded four datasets of NASH patients from the GEO database and merged them. After normalization, NASH group samples and 58 control group samples were obtained. To minimize the discrepancies between datasets, we first performed PCA on the raw data and visualized the results in a PCA plot (**Figure 2A**), which illustrates the variation across the four datasets. The initial PCA results revealed significant batch effects between the datasets. To mitigate these batch effects, we applied the ComBat method for batch effect correction and conducted a subsequent PCA analysis on the corrected data (**Figure 2B**). The PCA plot in **Figure 2B** demonstrates that, after batch effect correction, the samples from the four datasets are more tightly clustered in the lower-dimensional space, with a clearer separation between the NASH and control groups. The R “Limma” package was then used for analyzing the produced data. One thousand one hundred thirty-three differential genes were identified, including 562 up-regulated and 571 down-regulated genes. Volcano and heat maps were also drawn (**Figures 2C, D**). The volcano plot illustrates the relationship between the log fold change of each gene and the adjusted p-value, with significantly upregulated and downregulated genes highlighted in red and blue, respectively. The heatmap displays the expression patterns of the top 30 differentially expressed genes across different samples, providing further insights into the expression differences between the NASH and control groups.

### 3.2 WGCNA

**Figure 3:**
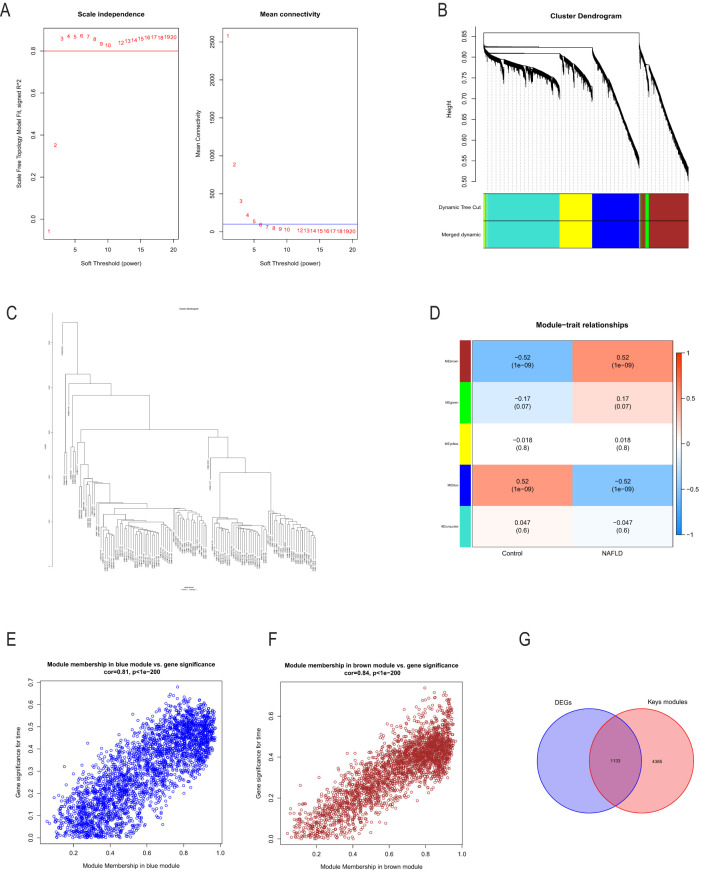
Using the WGCNA to screen essential modular genes in the integrated NASH dataset and the intersection of essential modular genes with DEGs to find critical NASH genes. The ideal β-value was found using a scale-free topology model, and β=3 was chosen as a soft threshold based on scale independence and average connectedness. **(B)** shows the gene dendrograms and the network tree diagrams of modular feature genes. **(C)** Presentation of samples of the clustered dendrograms. **(D)** A heatmap illustrating the connection between NASH status and genes with modular features. **(E, F)** Correlation plots between modular affiliation and gene significance. **(G)** Intersection of essential modular genes with DEGs taken through a Wayne diagram. WGCNA (weighted gene co-expression network analysis) of NASH.

To conduct a comprehensive analysis of the essential genes of NASH, we employed WGCNA to identify the gene modules most closely associated with NAFLD samples. By considering the scale independence and average linkage properties of the data, we set a soft threshold of 3 (as shown in **Figure 3A**), resulting in the generation of five modules. The MOD rule’s cluster tree diagram is depicted in **Figure 3B**, and the results of MOD rule sample clustering are presented in **Figure 3C**. Additionally, we analyzed the correlation between NASH and gene modules, as illustrated in **Figure 3D**. Our findings revealed a significant positive association between the brown module and NASH (r = 0.52, p = 1e-9) and a significant negative correlation between the blue module and NASH (r = -0.52, p = 1e-9). Consequently, we focused our investigation on the brown and blue modules. Furthermore, we observed a substantial association between gene importance and module affiliation in both the brown module (r = 0.84, p = 1e-200) and the blue module (r = 0.81, p = 1e-200), as shown in **Figures 3E and F**. These modules contained 5518 crucial genes significantly linked to NASH. To further identify essential genes in NASH, we intersected DEG genes with WGCNA essential genes in NASH samples, resulting in the retrieval of 1,133 genes for further research (**Figure 3G**).

### 3.3 DEGs and secreted proteins in COPD

**Figure 4:**
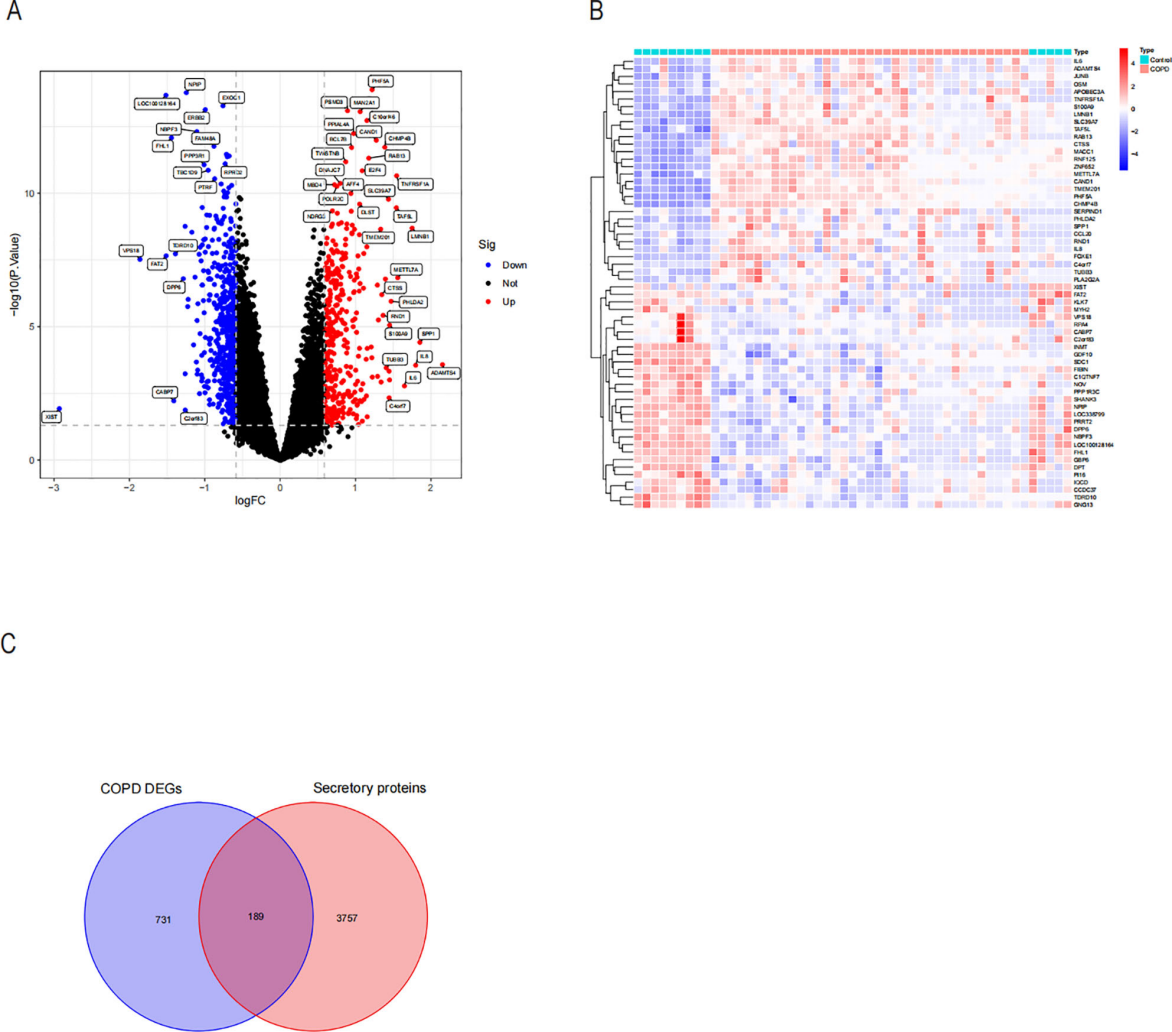
Differential expression analysis and identification of COPD-associated secreted proteins by differentially expressing the COPD dataset. **(A)** Volcano plot of revealed DEGs in the COPD dataset. **(B)** Heatmap of the top 30 DEGs in the COPD dataset that were up-regulated and down-regulated in their expression. **(C)** Intersection of the DEGs of COPD with genes of secreted proteins through the Weyen diagram. A total of 189 COPD-associated secreted proteins were identified.

Several studies have indicated a potential link between COPD and NASH, suggesting that COPD may contribute to the accelerated progression of NASH. This highlights the need to investigate pro-NASH genes involved in COPD (15, 28, 29). The COPD dataset comprises 37 COPD samples and 14 controls. We then used the “Limma” tool in R to run a differential analysis. In all, 920 genes with differential expression in COPD were found. Volcano maps and heat maps were plotted (**Figures 4A, B**). COPD involves a variety of inflammatory responses and cytokine changes, and we hypothesized that COPD may contribute to the progression of NASH through secreted proteins; we took the intersection of 920 differentially expressed genes and secreted proteins in COPD and obtained 189 secreted proteins associated with COPD (**Figure 4C**).

### 3.4 Network of PPI and functional enrichment of critical genes in NASH disease associated with COPD

**Figure 5:**
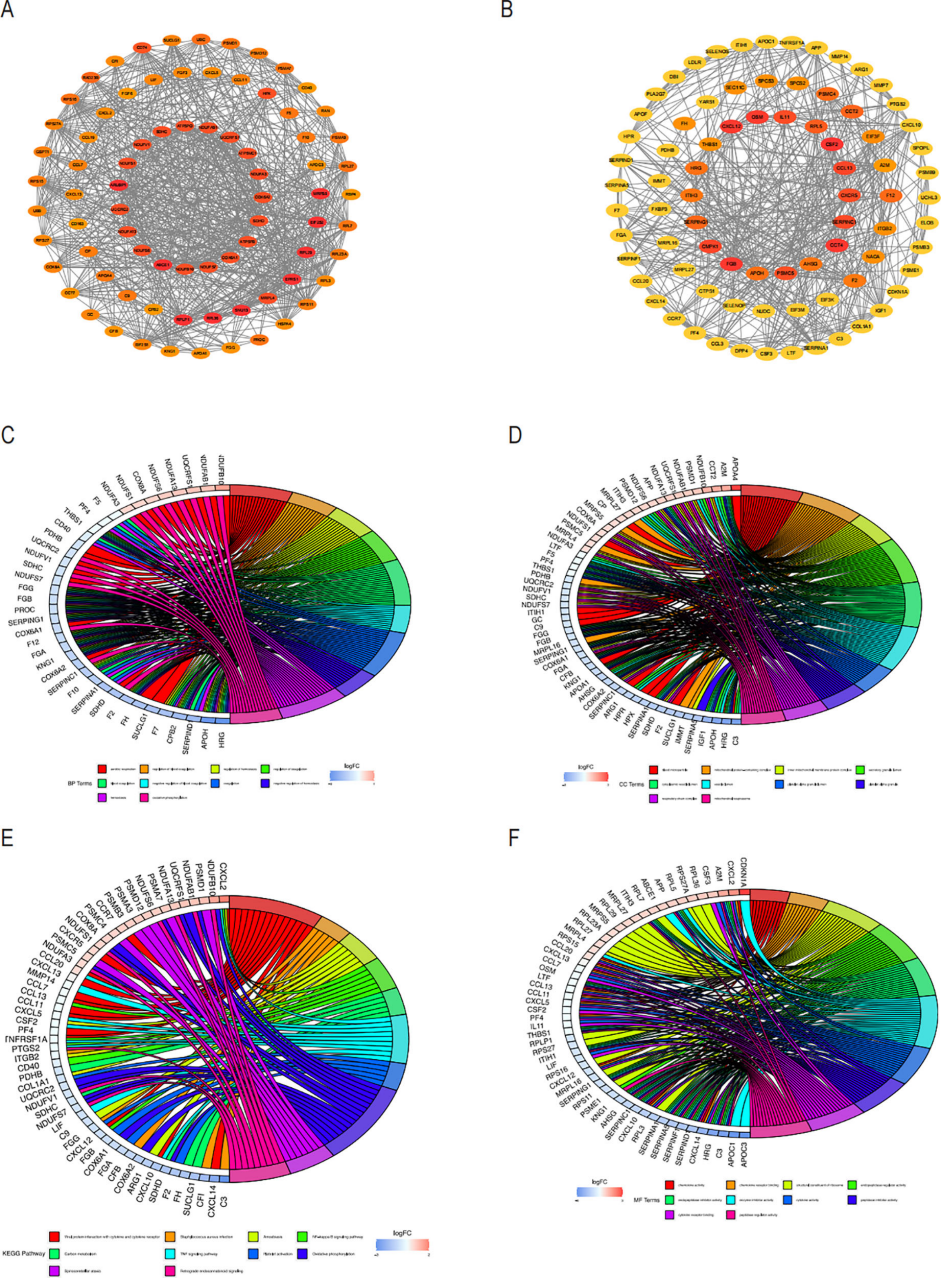
PPI analysis of COPD-associated secreted proteins with essential genes for NASH and node enrichment analysis for PPI screening. **(A)** PPI network of module1 genes based on the Cytoscape plugin scored top1 in MCODE analysis. Nodes are labeled as critical genes. **(B)** MCODE analysis of the PPI network of the top 2 scoring module genes. Circus displays the findings of the GO enrichment analysis of the genes included in Modules 1 and 2’s biological processes **(C)**, cellular components **(D)**, and molecular functions **(E)**. The **(F)** Circos plot displays the findings from the KEGG analysis of the genes involved in the PPI protein-protein interactions in modules 1 and 2.

We used the STRING database to analyze the interactions between the core gene proteins, studied the two most essential modules using the MCODE plug-in algorithm of Cytoscape software, and constructed a PPI network graph to identify the node genes (**Figures 5A, B**). We obtained the module genes of the essential modules and performed functional enrichment analysis. The results showed that the biological process (BP) of Aerobic respiration, regulation of blood coagulation, regulation of hemostasis, regulation of coagulation were associated with NASH (**Figure 5C**), while in cellular composition (CC) with blood microparticle, mitochondrial protein-containing complex, inner mitochondrial membrane protein complex (**Figure 5D**), and in MF with chemokine activity, chemokine receptor binding, structural constituent of ribosome (**Figure 5E**). The correlation between viral protein interaction with cytokine and cytokine receptors, the NF-kappa B signaling pathway, and the TNF signaling pathway was obtained by KEGG enrichment analysis (**Figure 5F**).

### 3.5 Identification of small molecule compounds

**Figure 6:**
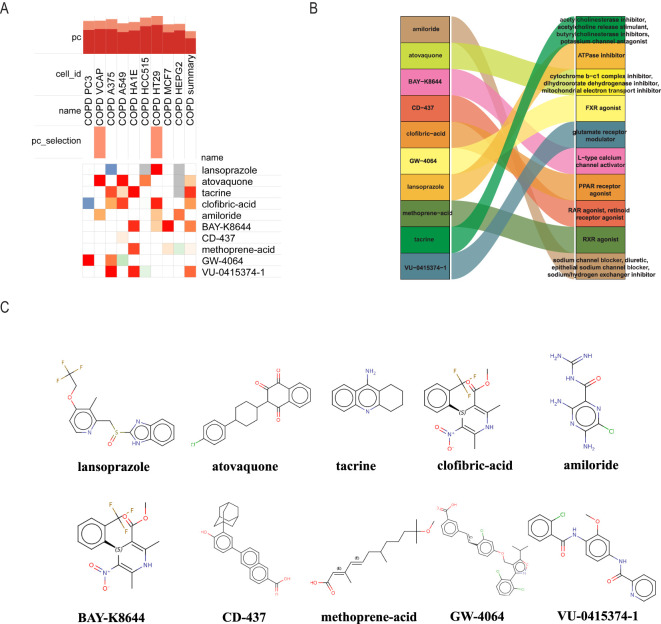
Using cMAP analysis to screen possible small molecule treatments for NASH. **(A)** The ten chemicals with the highest negative enrichment scores across 10 cell lines are displayed in a heat map based on cMAP analysis. **(B)** Sankey diagram showing the description of the ten compounds. **(C)** Displays the 10 compounds’ chemical structures.

We utilized the cMAP database to predict possible small-molecule drugs that could be therapeutic for COPD patients with NASH by importing up-regulated genes from pathogenic NASH-related genes. The top 10 highest-rated compounds from the study included lansoprazole, atovaquone, tacrine, clofibric-acid, amiloride, BAY-K8644, CD-437, methoprene-acid, GW-4064, VU-0415374-1, as drug candidates for the treatment of NASH (**Figure 6A**). The biological mechanisms and molecular architectures of these ten substances are illustrated. Comprehensive evaluation suggests that Amiloride and VU-0415374-1, as sodium ion channel inhibitors, may alleviate airway obstruction by improving airway fluid balance, while also exhibiting diuretic and sodium/hydrogen exchanger inhibitory properties. Clofibric-acid and GW-4064, as PPAR and FXR agonists, respectively, demonstrate significant anti-inflammatory and metabolic regulatory effects, indicating their potential in managing COPD-related inflammation and metabolic dysregulation. Atovaquone, through its role as a mitochondrial electron transport inhibitor, may reduce oxidative stress and mitigate cellular damage in severe COPD cases. Although Lansoprazole and Tacrine show limited direct efficacy in COPD, their mechanisms of action, such as glutamate receptor modulation and acetylcholinesterase inhibition, highlight their indirect therapeutic benefits, warranting further investigation. Future research should focus on preclinical validation of these drugs, deeper exploration of their molecular mechanisms, and evaluation of their efficacy in COPD models to facilitate clinical translation (**Figure 6B**). The chemical structures of the 10 selected drugs are presented to highlight their molecular characteristics, which are closely associated with their biological activities. For instance, the guanidine group in Amiloride is linked to its sodium channel blocking activity, while the carboxylic acid group in Clofibric-acid is essential for its anti-inflammatory effects. Although this study does not include experimental validation of these structures, their presentation provides a foundation for understanding their mechanisms of action and supports future research, such as molecular docking studies, structure-activity relationship (SAR) analyses, and the design of drug derivatives. These insights are expected to aid in the development of therapeutic strategies for COPD (**Figure 6C**).

### 3.6 Construction of a diagnostic model for COPD-associated NASH disease

**Figure 7:**
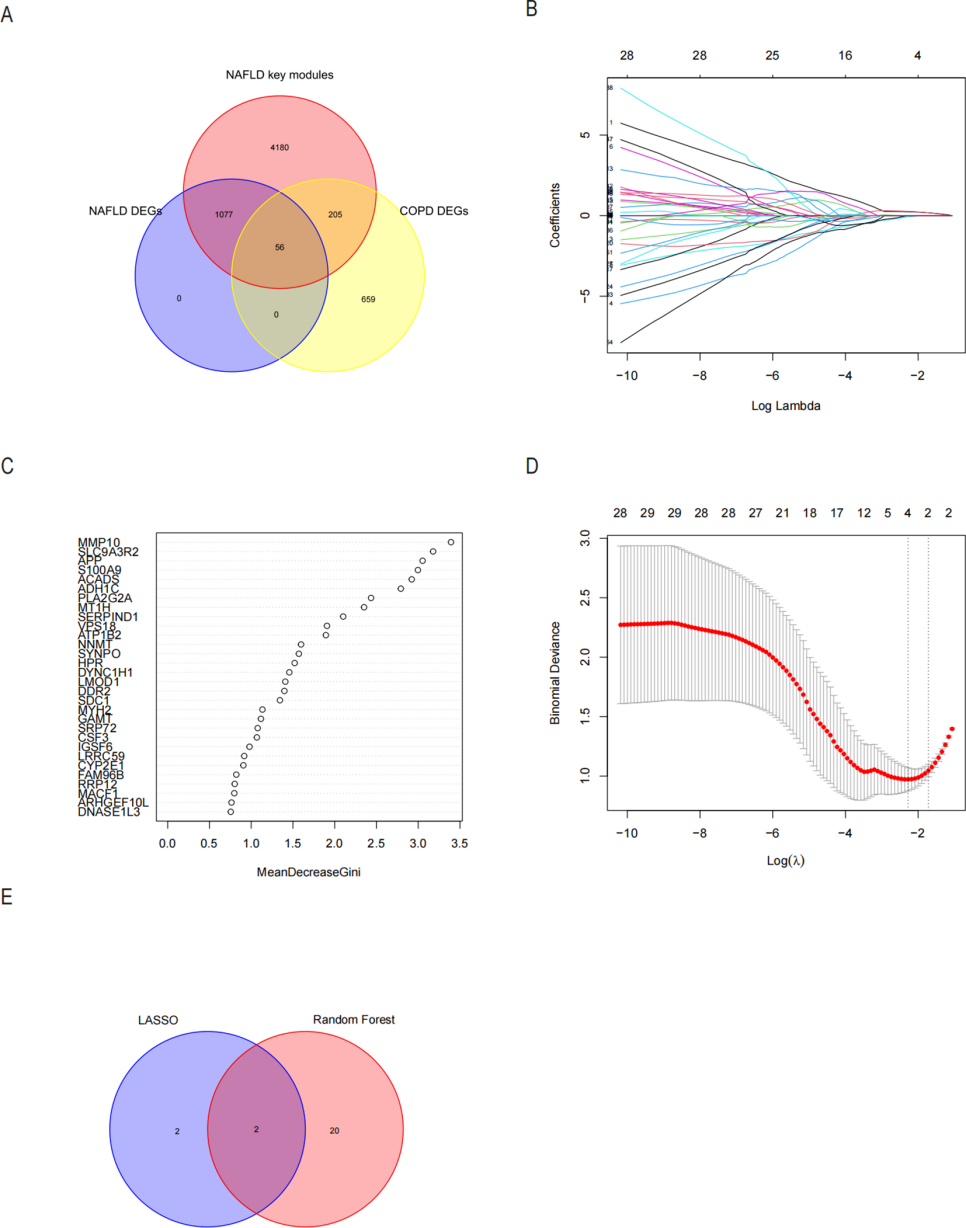
Possible diagnostic biomarkers for NASH linked to COPD to be found utilizing machine learning techniques. **(A)** A Venn diagram showing 56 genes for NASH-DEGs, key modules, and secretory proteins linked to COPD. **(B, C)** The LASSO logistic regression algorithm determined the Minimum and λ-values of the diagnostic biomarkers (n=4). **(D)** Based on the NASH in the 56 genes with MeanDecreaseGini scores greater than 1.0 for 22 biomarkers, the RF algorithm was selected. **(E)** Displays the Wayne diagram of the two genes that were found to be hub genes for COPD-associated NASH by the LASSO and RF algorithms. LASSO minimum absolute contraction and selection operators, RF random forests.

**Figure 8:**
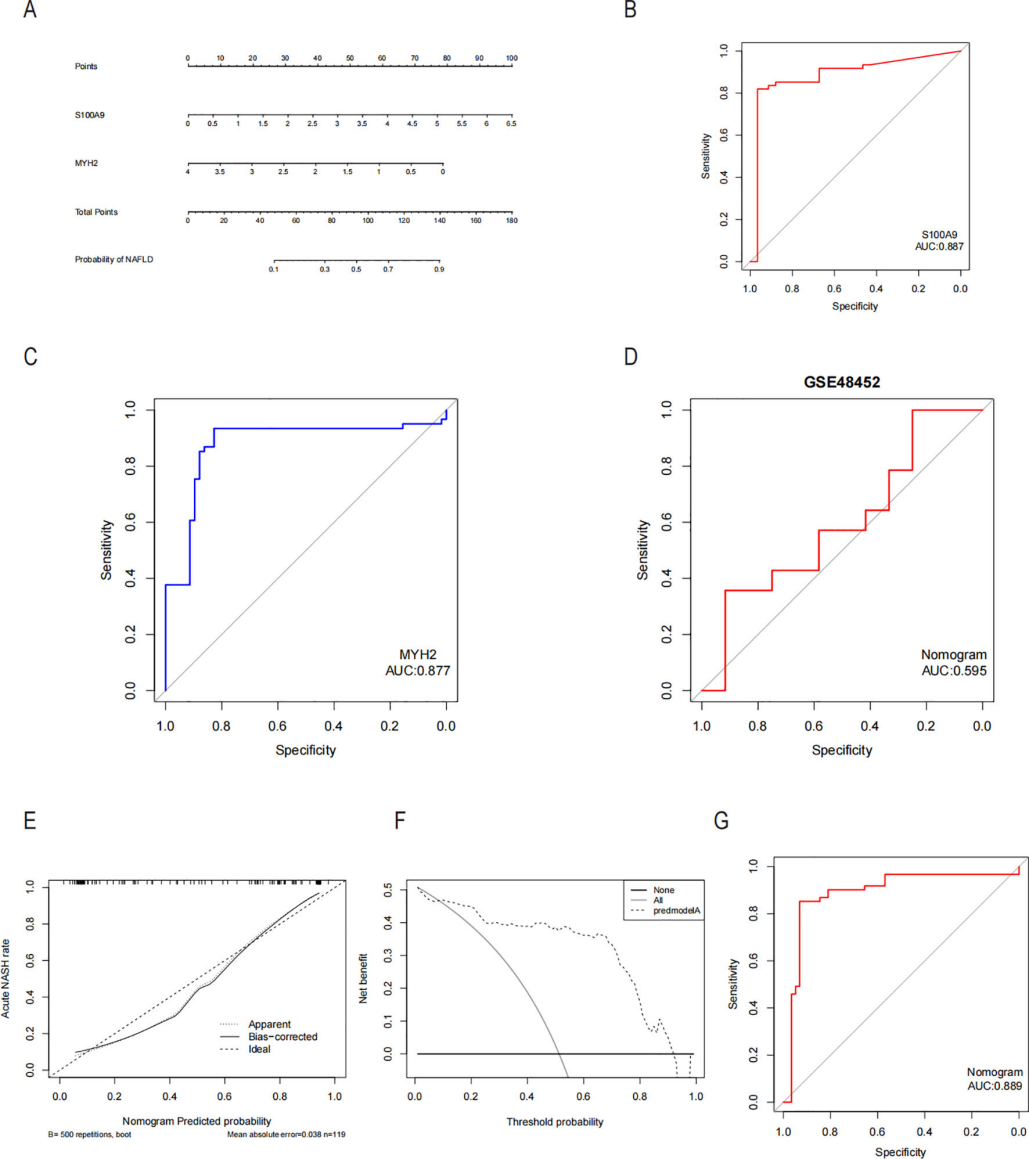
Diagnostic column-line diagram model construction and efficacy assessment. **(A)** Diagnostic biomarker-based column-line diagram construction. ROC curves for each candidate biomarker S100A9, MYH2, and diagnosis of COPD-associated NASH were plotted, and the column-line diagram model was constructed. **(B-D)** AUC values for each pivotal gene and the Nomogram were evaluated using the ROC. **(E)** Column-line diagram model predicting calibration curves for COPD-associated NASH. **(F)** DCA used for the column-line diagram model. **(G)** Our column line graph model predicts ROC curves for the diagnostic performance of NASH patients in the GSE48452 dataset in the GEO database. AUC Area under the curve, ROC subject operating characteristic curves, DCA decision curve analysis.

Since the critical genes of COPD-associated secretory proteins and NASH overlapped, which predicted that they might play crucial roles, 56 common vital genes were obtained between COPD-associated secretory proteins and NASH differential genes. The shared essential genes were used in the constructed diagnostic model of NASH (**Figure 7A**). Immediately after that, we performed LASSO regression analysis on the screened 56 common essential genes to identify four potential candidate genes (**Figures 7B, C**) and to screen the diagnostic markers more accurately; we also used the Random Forest (RF) machine learning algorithm to rank the 56 common genes according to the variable significance of each gene and to mention the MeanDecreaseGini>1 genes (**Figure 7D**). After overlapping 22 probable genes from RF and four probable genes from LASSO, only two hub genes—for S100A9 and MYH2—were found to overlap in two subgroups (**Figure 7E**). We constructed a nomogram based on logistic regression analysis using S100A9 and MYH2 as the hub genes (**Figure 8A**). The prediction ability of each crucial gene and the Nomogram model for COPD-associated NASH was assessed using the ROC and AUC. Sure enough, among them, the AUC value of S100A9 was 0.887, the AUC value of MYH2 was 0.877, and the AUC value of Nomogram was 0.889, suggesting that Nomogram has a robust diagnostic value for COPD-associated NASH disease (**Figures 8B–D**). The diagnostic model constructed by the Nomogram model had a prediction probability close to that of the ideal model (**Figure 8E**) and was analyzed by DCA to show its potential validity in the diagnosis of COPD-related NASH (**Figure 8F**). 12 samples of normal tissue and 14 samples from NASH patients were included in the GEO database’s GSE48452 dataset (**Figure 8G**), this Nomogram model also had some predictive value for COPD-combined NASH patients.

### 3.7 Analysis of immune cell infiltration and correlation between essential genes and immune cells in NASH patients

**Figure 9:**
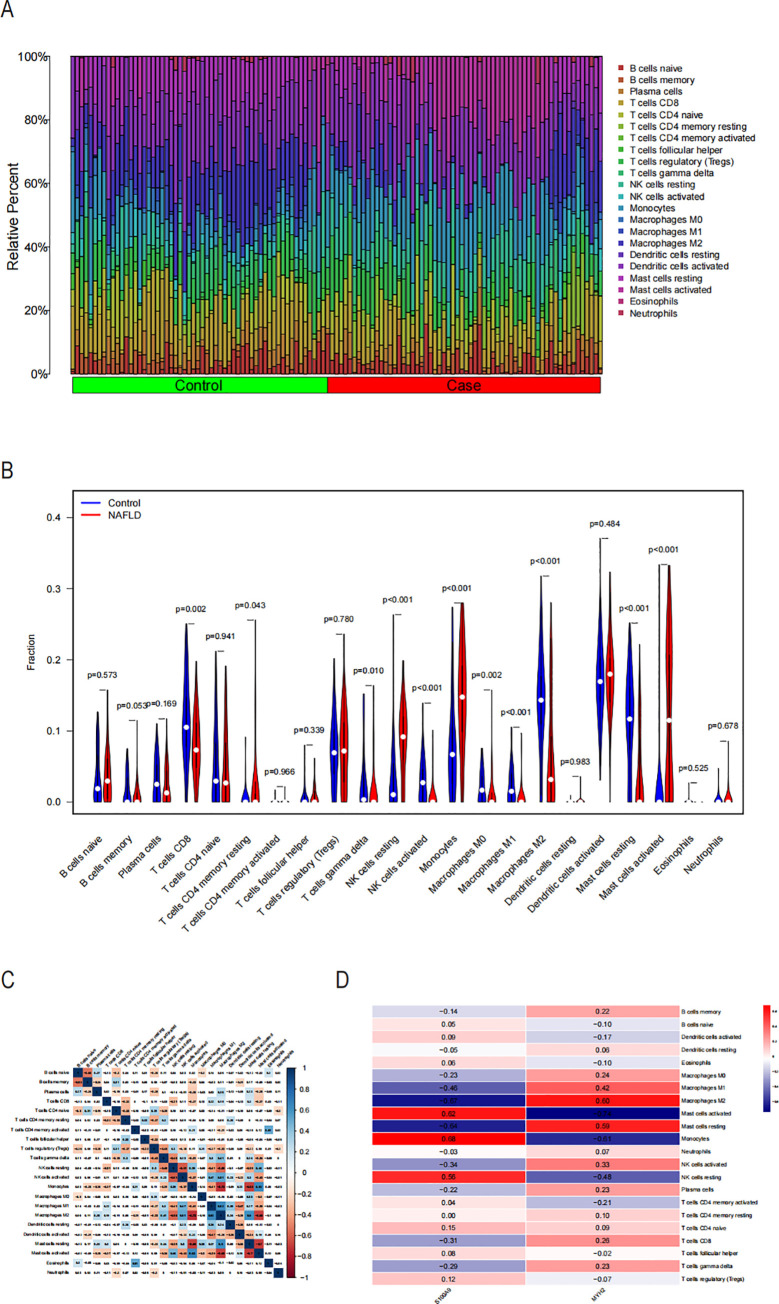
Examination of the invasion of immune cells in NASH. **(A)** The proportion of immune cells in the NASH and control groups is displayed using stacked histograms. **(B)** Violin graphs illustrating the 22 immune cells compared between the NASH and control groups. **(C)** Heatmap demonstrating a correlation between immune cells and infiltration of 22 immune cells at p<0.05 level. **(D)** Heatmap illustrating the relationship between two key genes and various infiltrating immune cells.

The inflammatory and immunological systems have a strong correlation with the pathogenic genes linked to COPD in NASH, according to functional and pathway studies. In order to investigate the immune regulatory mechanisms of NASH and the relationship between immune cell infiltration and diagnostic markers, the CIBERSORT algorithm was utilized to examine the characterization of NASH immune cells. **Figure 9A** displays the relative amounts of 22 immune cells in each sample. Significant variations were seen among the 10 subpopulations of immune cells, with activated Mast cells, Monocytes, resting NK cells, and CD4 memory T cells showing notable changes between NASH and control samples. The level of resting was elevated, but the levels of Mast cells resting, Macrophages M2, Macrophages M1, NK cells activated, T cells gamma delta, Macrophages M0, and T cells CD8 were reduced (**Figure 9B**). The correlation analysis of 22 types of immune cells revealed several significant correlations. There was a strong negative correlation (r=-0.61) between activated NK cells and resting NK cells. Additionally, there was a negative correlation (r=-0.73) between M2 macrophages and monocytes. On the other hand, there was a significant positive correlation (r=0.63) between activated mast cells and monocytes, as well as between resting mast cells and monocytes (r=-0.68). Furthermore, there was a significant positive correlation (r=-0.68) between activated mast cells and M2 macrophages, and between activated mast cells and resting mast cells (r=-0.7) (**Figure 9C**). **Figure 9D** shows the significant correlation of S100A9 and MYH2 genes with different immune cell types in NASH. It suggests that these two core genes may be involved in the immunoregulatory process of NASH by regulating the function or recruitment of specific immune cell types.

### 3.8 lncRNA-miRNA-mRNA network

**Figure 10:**
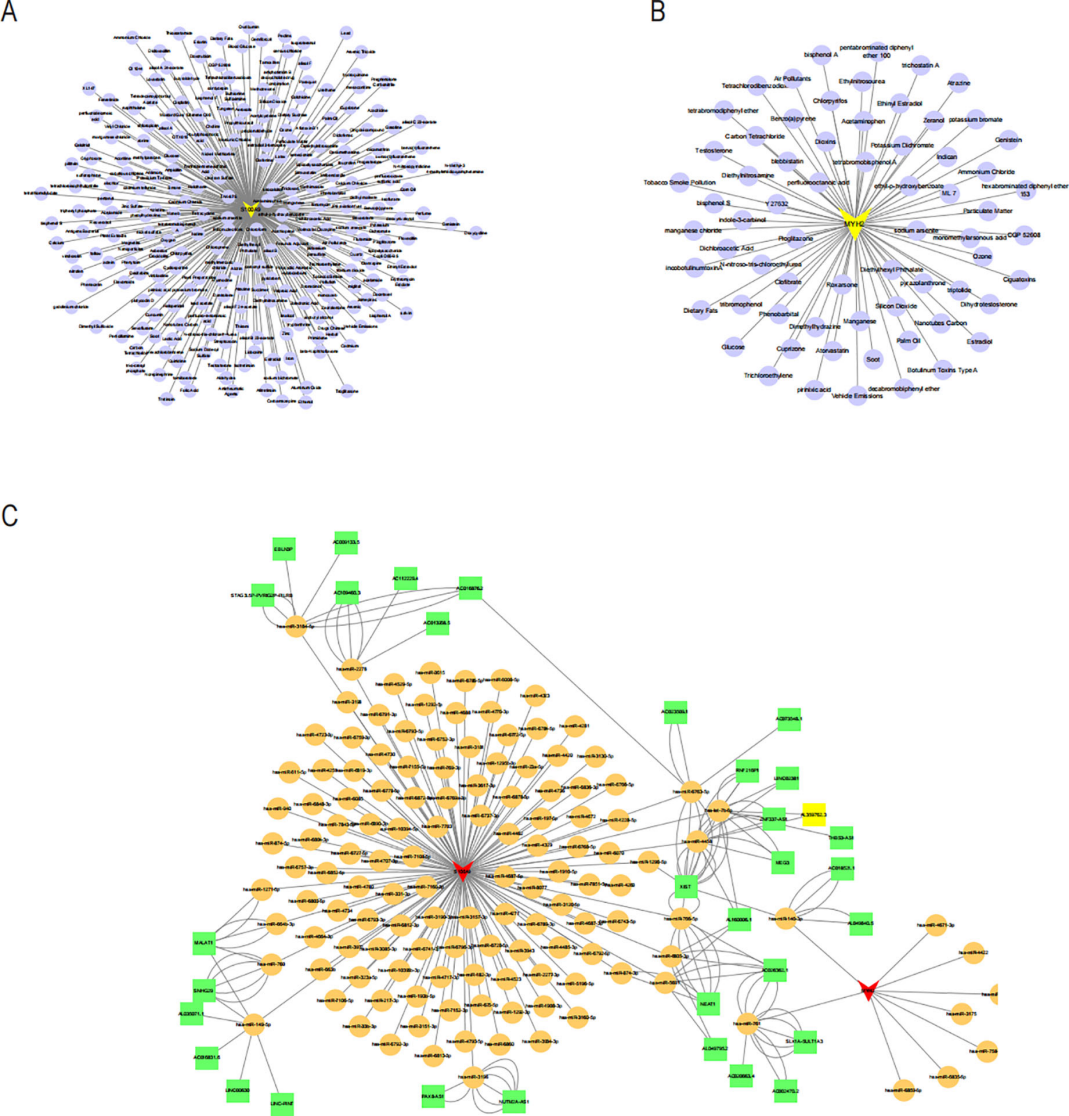
Drug-gene network of MYH2 **(A)** and S100A9 **(B)** lncRNA-miRNA-mRNA network was constructed. **(C)** Red triangles represent core genes. Yellow circles represent miRNAs, and green squares represent lncRNAs.

**Figure 11:**
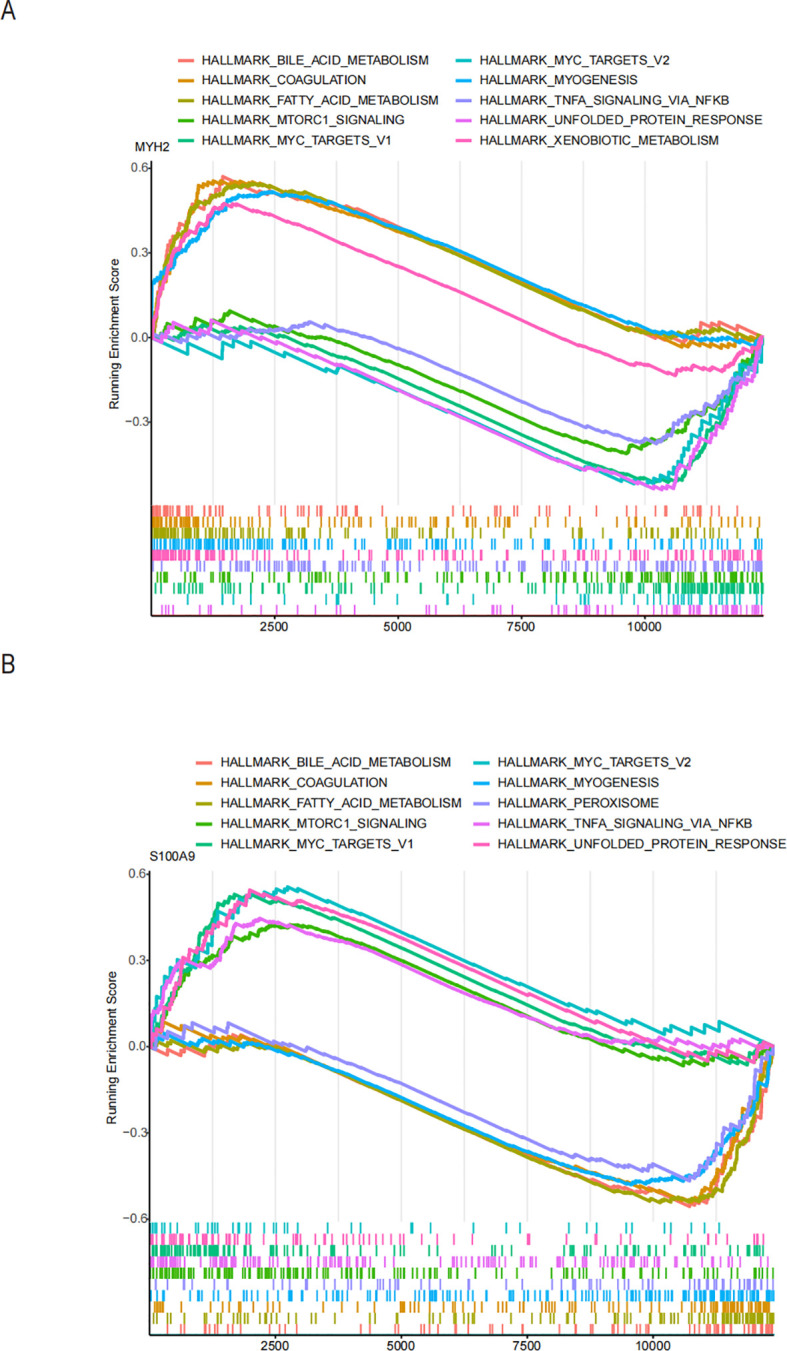
Functional enrichment analysis of the two hub genes using ssGSEA hallmark gene sets: **(A)** MYH2 and **(B)** S100A9.

To explore potential drugs for treating NAFLD in COPD patients, we searched the DGIdb database for potential drugs targeting the biomarkers. As shown in **Figures 10A, B**, 67 drugs targeting MYH2 and 254 drugs targeting S100A9 were mined. Meanwhile, the lncRNA-miRNA-mRNA network was constructed by predicting the target miRNAs of the biomarkers and further predicting the lncRNAs targeting the miRNAs (**Figure 10C**). GSEA enrichment analysis showed (**Figures 11A, B**) that MYH2 and S100A9 were mainly enriched in the following: “HALLMARK_FATTY_ACID_METABOLISM “,”HALLMARK_TNFA_SIGNALING_VIA_NFKB. “

### 3.9 Validation of the screened hub genes

**Figure 12:**
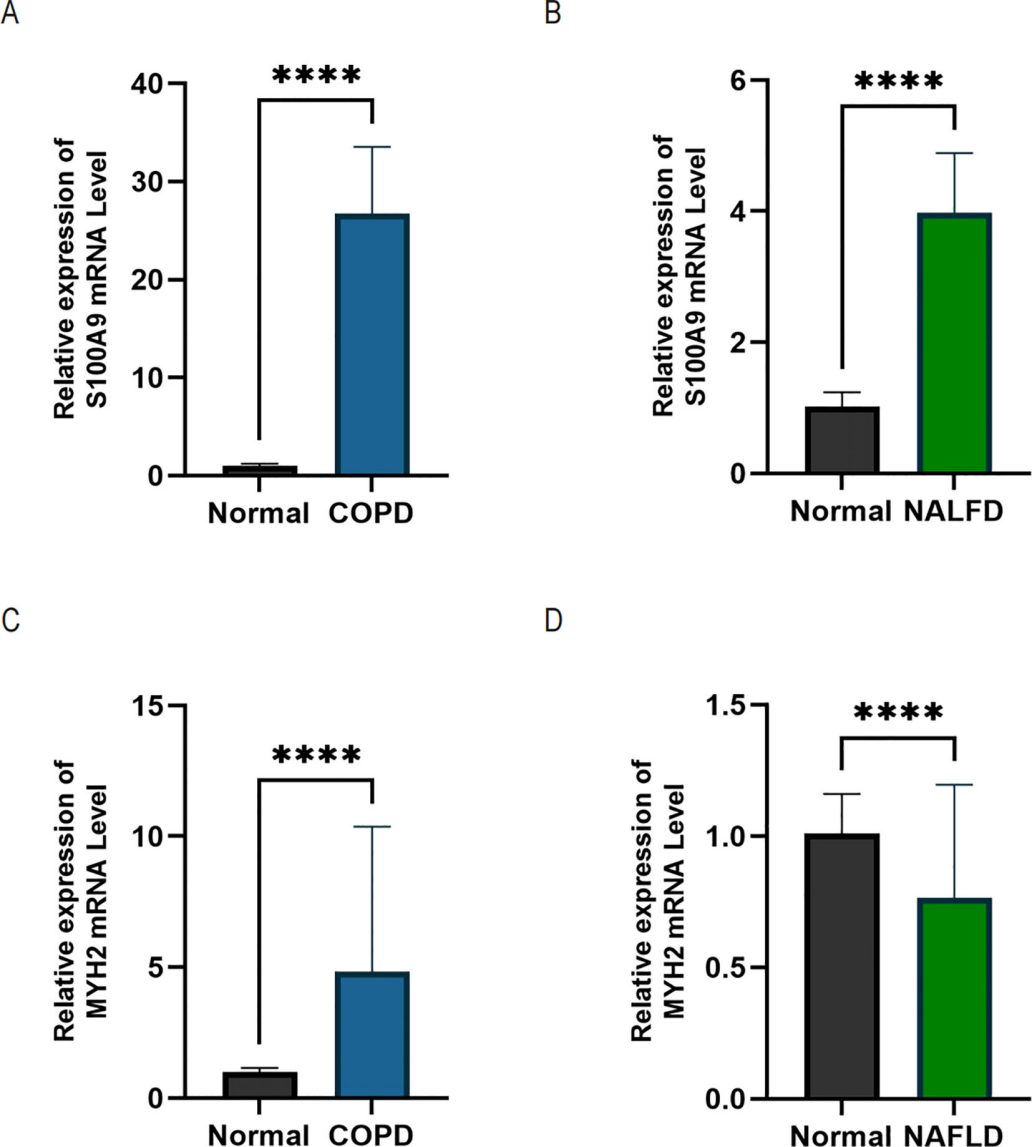
qRT-PCR analysis was performed using serum samples from healthy individuals, NAFLD patients, and COPD patients. **(A, B)** Relative expression levels of S100A9 mRNA in COPD and NAFLD patients. **(C, D)** Relative expression levels of MYH2 mRNA in COPD and NAFLD patients. Data are expressed as mean ± SD (n = 25). Statistical comparisons between groups were performed using Student's t-test with Welch’s correction. ****p < 0.0001 indicates a statistically significant difference.

In this study, we screened the pivotal genes S100A9 and MYH2to validate the accuracy of the bioinformatics approach. We collected 25 serum samples each from healthy individuals, COPD patients, and NAFLD patients with the informed consent of volunteers. qRT-PCR was performed to validate the critical genes at the mRNA level. The results demonstrated that S100A9 expression was significantly upregulated in both COPD (**Figure 12A**) and NAFLD patients (**Figure 12B**). In contrast, MYH2 expression was markedly increased in COPD patients (**Figure 12C**) but showed a notable downregulation in NAFLD patients (**Figure 12D**).

## 4 Discussion

NAFLD and COPD are significant public health issues characterized by a high incidence of illness and death, as well as significant financial burdens (28). An increasing number of studies have suggested a potential link between the two diseases. Authoritative epidemiological and clinical evidence further indicates that patients with COPD have a significantly higher prevalence of NAFLD compared to the general population with similar pathogenic backgrounds and high co-morbidities. This association is not coincidental but is attributed to their shared pathophysiological mechanisms. Viglino D indicated that both COPD and NAFLD involve inflammatory processes, which may also promote the progression of NAFLD (15).

We studied these two diseases using bioinformatics methods with the help of public databases. This can facilitate our comprehension of the progression of both diseases and offer novel insights for diagnosis and therapy. KEGG enrichment analyses and GO-biological process annotation indicated that the inflammatory-immune pathway may be vital in developing COPD-associated NASH. Comprehensive evaluation suggests that Amiloride and VU-0415374-1, as sodium ion channel inhibitors, may alleviate airway obstruction by improving airway fluid balance, while also exhibiting diuretic and sodium/hydrogen exchanger inhibitory properties. Clofibric-acid and GW-4064, as PPAR and FXR agonists, respectively, demonstrate significant anti-inflammatory and metabolic regulatory effects, indicating their potential in managing COPD-related inflammation. Atovaquone, through its role as a mitochondrial electron transport inhibitor, may reduce oxidative stress in severe COPD cases. While Lansoprazole and Tacrine show limited direct efficacy, their mechanisms, such as glutamate receptor modulation and acetylcholinesterase inhibition, suggest indirect therapeutic benefits. The inclusion of chemical structures provides a basis for understanding the molecular characteristics underlying these effects, such as the guanidine group in Amiloride for sodium channel blocking and the carboxylic acid group in Clofibric-acid for anti-inflammatory activity. Although experimental validation of these structures is not included, they offer insights for future molecular docking, SAR studies, and derivative design, supporting the development of therapeutic strategies for COPD.

Liver biopsy remains the gold standard for diagnosing NASH, but its invasive nature limits its clinical applicability. This underscores the urgent need for reliable blood-based biomarkers as non-invasive diagnostic tools for NASH (30). This study utilized comprehensive bioinformatics methods to identify S100A9 and MYH2 as potential biomarkers from public databases and further validated their diagnostic value through serum sample analysis from COPD and NAFLD patients. In the ROC analysis based on public datasets, S100A9 and MYH2 demonstrated excellent diagnostic performance, with AUC values of 0.887 and 0.877, respectively, indicating high sensitivity and specificity. Additionally, a nomogram model integrating S100A9 and MYH2 further improved diagnostic performance, achieving an AUC of 0.889, providing a theoretical foundation for risk assessment and screening of COPD-related NAFLD.

In the serum validation analysis, S100A9 levels were significantly elevated in the serum of both COPD and NAFLD patients, supporting its potential as a systemic inflammatory biomarker. In contrast, MYH2 was significantly upregulated in the serum of COPD patients, whereas it was downregulated in NAFLD patients. This discrepancy may be attributed to the distinct pathological mechanisms underlying the two diseases. In NAFLD, the downregulation of MYH2 may be related to disease-specific mechanisms such as insulin resistance and lipid metabolism dysregulation. On the other hand, the increased expression of MYH2 in COPD patients may reflect a disease-specific adaptive remodeling of skeletal muscle, supporting its role as a complementary diagnostic marker for COPD-related NAFLD (31–33).

By combining bioinformatics screening and serum-based validation, this study confirmed the potential diagnostic value of S100A9 and MYH2, providing a scientific basis for further exploration of their functional mechanisms and clinical applications. Future studies should integrate tissue samples and functional experiments to refine the application of these biomarkers in the diagnosis and treatment of COPD-related NAFLD.

In this study, we focused on S100A8/S100A9 and explored their roles in NAFLD and COPD. Previous research by Averill et al. found that in low-density lipoprotein receptor (LDLR)-deficient S100A9 chimeras, insulin resistance did not improve after a high-fat diet (34). Furthermore, S100A8 can bind to receptors such as Toll-like receptor 4 (TLR4) and receptor for advanced glycation end-products (RAGE), activating downstream signaling pathways and acting as a chemokine to recruit macrophages and neutrophils, thereby exacerbating hepatic inflammation (35). Notably, S100A9 deletion is often accompanied by reduced S100A8 expression, suggesting a potential regulatory relationship that warrants further investigation. S100A9 has also been shown to bind to TLR4 and activate the NF-κB pathway through a MyD88-dependent mechanism, inducing the production of inflammatory factors (36, 37). Liu et al. demonstrated that S100A9 levels in rat liver and serum were consistent with hepatic mRNA levels, suggesting its potential as a biomarker for predicting NAFLD progression and distinguishing between phenotypes (38). In COPD, S100A9 is strongly expressed in immune cells in the lungs and contributes to disease progression by stimulating neutrophil adhesion and activating NF-κB via TLR4. Importantly, inhibiting S100A9 has been shown to significantly reduce neutrophil-associated inflammation in COPD lungs (39). Consistent with our findings, S100A9 plays a critical role in both NAFLD and COPD, underscoring its potential as a key target for developing diagnostic and therapeutic strategies for NASH in COPD patients.

The essential role of MYH2, which encodes myosin heavy chain IIA, in the pathophysiology of myopathies and cachexia has been well-documented in numerous studies (26, 40). Cachexia is a prevalent comorbidity in chronic conditions such as COPD and NAFLD. It is frequently associated with systemic inflammation, characterized by elevated levels of TNF-α and IL-6, and metabolic dysregulation, including insulin resistance. These factors may collectively influence MYH2 expression, contributing to anabolic resistance and the degradation of muscle fibers. In COPD, chronic inflammation and hypoxic conditions may promote adaptive remodeling of fast-twitch muscle fibers through MYH2 regulation. Notably, data from the 2008-2011 Korean National Health and Nutrition Examination Survey indicate that COPD patients with sarcopenia are more likely to be comorbid with NAFLD, suggesting a potential link between muscle wasting and metabolic dysfunction in these populations (41). Conversely, systemic insulin resistance and inflammation induced by NAFLD may indirectly affect MYH2 expression and skeletal muscle function (42, 43).The role of MYH2 in muscle metabolism, inflammatory responses, and chronic diseases underscores its potential as a therapeutic target.

In conclusion, this study demonstrates that the differential expression patterns of S100A9 and MYH2 in COPD and NAFLD have significant diagnostic potential. S100A9 serves as a cross-disease biomarker, reflecting a shared inflammatory response, and thus shows promise in diagnosing both diseases. On the other hand, MYH2 exhibits disease-specific expression changes, effectively distinguishing COPD from NAFLD. The combined use of these two biomarkers in a diagnostic model not only improves early diagnostic accuracy but also enhances the differentiation between the two diseases. Therefore, the joint application of S100A9 and MYH2 provides a novel approach for the clinical diagnosis of COPD and NAFLD, with important implications for early detection and disease monitoring. Further clinical validation is crucial to confirm the diagnostic efficacy and real-world applicability of these biomarkers. The potential for improved diagnostic tools, based on S100A9 and MYH2, could lead to more accurate disease differentiation and timely interventions. Importantly, while this study focuses on diagnostic markers, previous research has shown that managing NAFLD in COPD patients may improve patient outcomes and survival (44). Therefore, integrating NAFLD screening in COPD patients may not only enhance early diagnosis but also contribute to better disease management and prognosis.

### 4.1 Limitations

However, despite our efforts to combine numerous datasets, this study still has certain constraints, primarily due to the restricted size of our sample. While bioinformatics tools have identified vital genes mostly linked to immunity and inflammation, further research is required to understand how these crucial genes regulate immune cells. Despite an extensive literature review revealing substantial evidence of a potential association between MYH2 and COPD as well as NAFLD, no studies to date have demonstrated a direct role for MYH2 in these conditions. Moreover, our research faced limitations in resources and data availability, precluding a detailed analysis of the correlation between MYH2 expression and inflammatory markers or direct experimental validation using muscle tissue samples. We acknowledge that these constraints have limited our ability to comprehensively elucidate the role of MYH2 in COPD and NAFLD.

## Data Availability

The diverse datasets utilized in this investigation, including GSE24807, GSE48452, GSE66676, GSE63067, GSE38974, and GSE106986, are available for download from internet databases.
